# microRNA-122 Dependent Binding of Ago2 Protein to Hepatitis C Virus RNA Is Associated with Enhanced RNA Stability and Translation Stimulation

**DOI:** 10.1371/journal.pone.0056272

**Published:** 2013-02-06

**Authors:** K. Dominik Conrad, Florian Giering, Corinna Erfurth, Angelina Neumann, Carmen Fehr, Gunter Meister, Michael Niepmann

**Affiliations:** 1 Institute of Biochemistry, Faculty of Medicine, Justus-Liebig-University, Giessen, Germany; 2 Institute of Biochemistry, Faculty of Biology and Preclinical Medicine, University of Regensburg, Regensburg, Germany; Drexel University College of Medicine, United States of America

## Abstract

Translation of Hepatitis C Virus (HCV) RNA is directed by an internal ribosome entry site (IRES) in the 5′-untranslated region (5′-UTR). HCV translation is stimulated by the liver-specific microRNA-122 (miR-122) that binds to two binding sites between the stem-loops I and II near the 5′-end of the 5′-UTR. Here, we show that Argonaute (Ago) 2 protein binds to the HCV 5′-UTR in a miR-122-dependent manner, whereas the HCV 3′-UTR does not bind Ago2. miR-122 also recruits Ago1 to the HCV 5’-UTR. Only miRNA duplex precursors of the correct length stimulate HCV translation, indicating that the duplex miR-122 precursors are unwound by a complex that measures their length. Insertions in the 5′-UTR between the miR-122 binding sites and the IRES only slightly decrease translation stimulation by miR-122. In contrast, partially masking the miR-122 binding sites in a stem-loop structure impairs Ago2 binding and translation stimulation by miR-122. In an RNA decay assay, also miR-122-mediated RNA stability contributes to HCV translation stimulation. These results suggest that Ago2 protein is directly involved in loading miR-122 to the HCV RNA and mediating RNA stability and translation stimulation.

## Introduction

Hepatitis C Virus (HCV) is the sole member of the genus Hepacivirus in the positive strand RNA virus family *Flaviviridae*. HCV replicates preferentially in the liver, and all steps of the replication cycle take place exclusively in the cytoplasm of the infected cell where the positive strand HCV RNA genome directly serves as a template for translation of the viral gene products [1]. In contrast to most cellular mRNAs, the initiation of translation of the HCV RNA is directed by an internal ribosome entry site (IRES) element that is located in the viral RNÁs 5′-untranslated region (5′-UTR). This IRES recruits the ribosomes to the internal translation start site on the viral RNA [2], [3].

**Figure 1:**
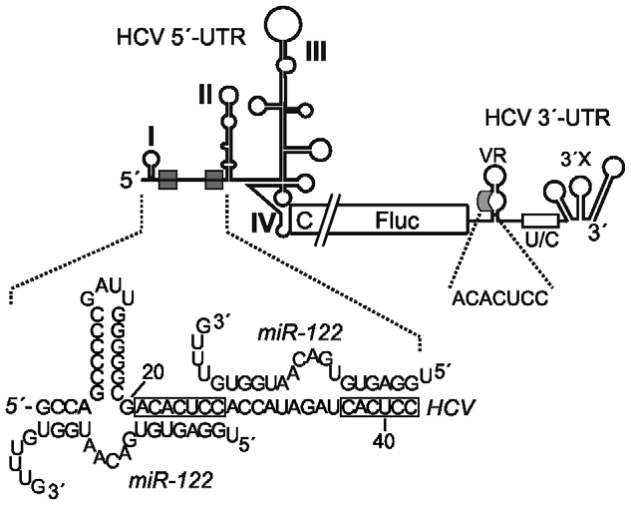
The HCV reporter RNA. The HCV untranslated regions (UTRs), shown in the context of the HCV reporter RNA with the complete HCV 5′-UTR, partial core coding sequence (C), the firefly luciferase (Fluc) reporter gene and the HCV 3′-UTR with the variable region (VR), the poly(U/C)-tract (U/C) and the 3′-X region. The miR-122 seed target sites are shown as grey boxes, and the putative binding of miR-122 to the HCV 5′-terminal sequences is shown in the enlargement. The enlargement from the 3′-UTR shows the conserved ACACUCC sequence in the otherwise variable region.

The HCV 5′-UTR contains four RNA stem-loop structures (see Fig. 1). Stem-loops I and II are involved in RNA replication. Partially overlapping, stem-loops II through IV constitute the IRES element. The activity of the HCV IRES is stimulated by the 3′-UTR of the viral RNA [4], [5], [6], ensuring efficient translation only of undegraded full-length viral RNAs that are competent for virus progeny production. The HCV IRES can bind to the sole ribosomal 40S subunit in the absence of any eukaryotic translation initiation factor (eIF) by means of the IRES RNA structures including the base of stem-loop III and stem-loop IV [7]. Subsequent initiation steps require the binding of eIF3 to the apical regions of stem-loop III [8], while HCV translation initiation is independent of eIF4 group factors [9]. In addition, several other RNA-binding proteins modulate HCV IRES activity [2], [3]. While the expression of cellular surface receptors involved in HCV binding and entry is not strictly limited to hepatocytes [10], a contribution to tissue selectivity can also be attributed to the stimulation of HCV translation and genome accumulation by microRNA-122 (miR-122) [11], [12], [13], [14] since this microRNA is expressed preferentially in liver cells or in the HuH-7 hepatoma cell line [15], [16], [17], [18].

microRNAs (miRNAs) regulate eukaryotic gene activity at the post-transcriptional level [19], [20]. Processing of miRNA precursors results in ∼ 22 bp miRNA duplexes with 3′-overhangs. This miRNA duplex is then unwound, and the strand with its 5′-end at the thermodynamically less stable end of the duplex is retained in a microRNA/protein (miRNP) complex as a sequence-specific guide to find its target mRNA by base-pairing, while the opposite (passenger) strand is discarded. In the complex, the miRNA guide strand is positioned with its 3′-end in the PAZ domain and with its 5′-end between the MID and PIWI domains of an Argonaute (Ago) protein [21], [22], thereby making the so-called seed region near the miRNÁs 5′-end (usually miRNA nucleotides 2 to 8) accessible for base-pairing with the target sequence in the mRNA. Also other regions of the miRNA may be available for base-pairing with the target [23]. The effector function of this Ago-containing complex then depends on the extent of base-pairing between small RNA and mRNA target. When the small RNA matches perfectly to its target, an RNA induced silencing complex (RISC) forms, and the RISC containing an Ago2 protein cleaves the target mRNA opposite to the guide strand. In contrast, when base pairing between miRNA and target mRNA is imperfect, the interaction of the miRNA-protein (miRNP) complex with the target mRNA usually results in translation repression.

Between the stem-loops I and II of the highly conserved 5′-UTR of HCV, there are two sequences complementary to the seed sequence of miR-122 (Fig. 1), a 7-nucleotide sequence (ACACUCC) and a 6-nucleotide sequence (CACUCC). In the human hepatoma cell line HuH-7, HCV genome accumulation is enhanced by miR-122 binding to these target sites in the HCV 5′-UTR [11]. Specifically, we have shown that interaction of miR-122 with these target sites stimulates HCV translation in HuH-7 and HeLa cells as well as in the rabbit reticulocyte lysate in vitro-translation system [12], [13], and the interaction between HCV RNAs with a mutated miR-122 target site and a correspondingly mutated miR-122 led to a strong increase in ribosome association in HeLa cells [12]. Besides detailing the probable tissue-specific action of miR-122 on the HCV RNA, this was one of the first reports of translation stimulation by microRNAs [12], [24], [25]. In addition to these two miR-122 target sites in the HCV 5′-UTR, there is a highly conserved sequence in the otherwise variable region of the HCV 3′-UTR (see Fig. 1). This sequence (ACACUCC) is similar to the miR-122 seed target consensus sequences in the 5′-UTR, but no function of this sequence in miR-122 action on HCV propagation was demonstrated so far [11], [12].

Moreover, in the HCV 5′-UTR a sequence comprising part of the two miR-122 target sites and the nucleotides between them can hybridize to a sequence stretch in the Core protein coding region, thereby resulting in translation inhibition [26], [27], [28]. In structural terms, this long-range RNA-RNA interaction can switch the conformation of the HCV 5′-UTR from an "open" to a "closed" conformation, and this structural switch was shown in vitro to be relieved by the single-stranded mature miR-122 guide strand that interferes with the long-range interaction [29]. Recently, we have confirmed the functional implications of the above structural study by showing that single-stranded miR-122 as well as actually any single-stranded RNA oligonucleotide that can interfere with the inhibitory long-range RNA-RNA interaction stimulates translation of a HCV reporter RNA in reticulocyte lysate [30].

However, only single-stranded miR-122 guide strand but not duplex miR-122 precursors were found to stimulate HCV translation in reticulocyte lysate, whereas in HuH-7 and HeLa cells only duplex miR-122 precursors stimulate HCV translation [30]. These findings suggest that the miRNA precursor processing events that occur in cells are obviously either missing or different in the reticulocyte lysate. In cells, stimulation of HCV translation requires duplex miR-122 precursors, indicating that a cellular machinery unwinds the duplex miRNA precursors and only then uses the miRNA guide strand as a probe to act on the target mRNA, a process which is reminiscent of microRNA/protein complexes containing an Ago protein [19], [20].

Here we show that miR-122 enhances HCV translation efficiency in cells only when supplied as duplex miR precursor, of which precursors of the correct length of 22 nucleotides work best. Consistent with the hypothesis that miRNP complexes containing an Ago protein are involved in unwinding the duplex miRNA precursor and using the mature microRNA guide strand as a probe, we show that Ago2 protein is directly involved in the complex with the miR-122 that binds the HCV 5′-UTR, whereas such a complex does not form with the consensus sequence in the 3′-UTR. The results of an RNA decay assay suggest that both RNA stability and translation stimulation contribute to enhanced HCV translation. Partially masking the miR-122 binding sites in a stem-loop structure impairs translation stimulation by miR-122, indicating that access of the miRNP complex to the HCV 5′-UTR requires that the miR-122 target sites are provided in a largely single-stranded RNA region.

## Results

### Ago2 protein binds to the HCV 5′-UTR in a miR-122 dependent manner

Recently we have shown that duplex miR-122 of 22 nt length is effective in stimulating HCV translation in cells [30], suggesting that Ago proteins confer the stimulatory action of miR-122 on HCV translation. For mediating the microRNA guide function to find the target sequences, Ago proteins unwind duplex miRNA precursors of defined length and then use the single-strand microRNA guide strand by holding the microRNA with its 5′-phophate in a pocket in their MID domain and its 3′-end in the PAZ domain. Thereby the seed sequence (nucleotides 2 to 8 of the miRNA) as well as other internal sequences of the miRNA are exposed for hybridization with the target mRNA [21], [22].

**Figure 2:**
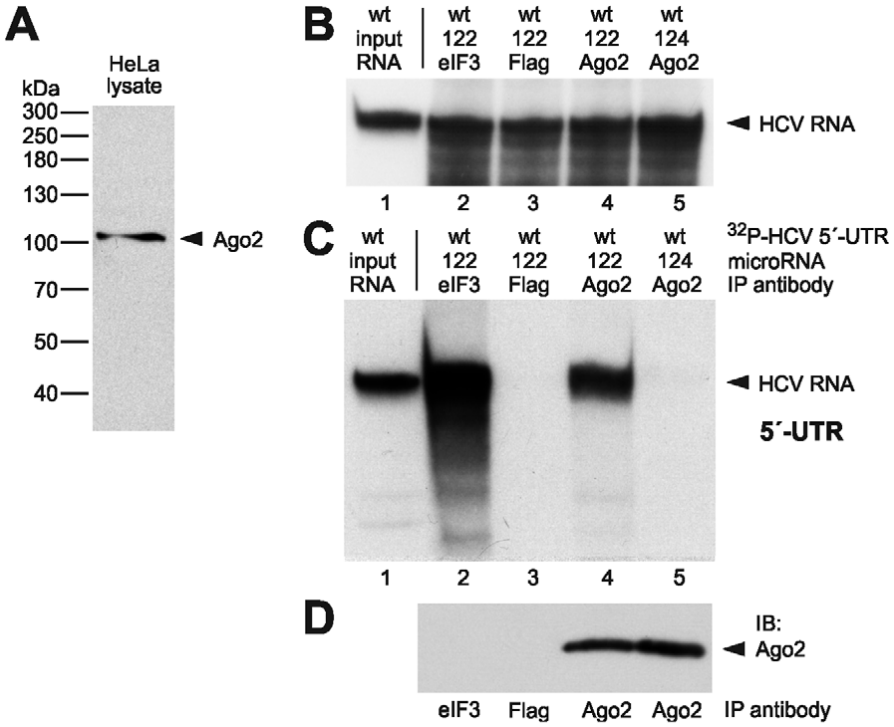
Ago2 protein interacts miR-122-dependently with the HCV 5′-UTR. (A) Immunoblot of HeLa cell lysate with the anti-Ago2 antibody monoclonal 11A9 [32]. (B–D) Analysis of ^32^P-labelled HCV 5′-UTR RNA after transfection into HeLa cells in the presence or absence of miR-122 or control miR-124 duplexes as indicated. The cells were lysed 6 h after transfection. (B) Aliquots of the cell lysate were used for RNA re-extraction to check the integrity of the input RNA prior to immunoprecipitation. The re-extracted radioactive HCV RNA was visualized after gel electrophoresis and autoradiography. A quantification of the RNA amounts in lanes 2–5 is shown in Fig. S1A. (C) Immunoprecipitation (IP) of Ago2-HCV 5′-UTR RNA complexes. RNA-Ago2 protein complexes were immunoprecipitated from the cell lysates with anti-Ago2 antibodies. From the precipitate, RNA was re-extracted, and the radiolabelled HCV RNA was visualized after gel electrophoresis and autoradiography. In lane 1, the input RNA is shown (1∶100 dilution). In lane 2, an anti-eIF3 antibody was used as a positive control. In lane 3, an anti-Flag antibody was used as a negative control. (D) Aliquots of the immunoprecipitates used in lanes 2–5 in (C) were checked for Ago2 in an immunoblot.

In order to test if Ago2 protein associates with the HCV RNA, we performed immunoprecipitations of protein complexes formed with the HCV reporter RNAs in cells. To this end, HeLa cells were transfected with the ^32^P-labelled HCV 5′-UTR RNA in the presence or absence of 22 nt duplex microRNAs. 6 hours after transfection, the cells were lysed, and Ago2 protein containing miRNP complexes were immunoprecipitated from the cytoplasmic fraction with an Ago2-specific monoclonal antibody [31], [32]. This antibody recognizes the Ago2 protein in the immunoblot without any visible cross-reaction to other proteins (Fig. 2A). In the presence of miR-122, the anti-Ago2 antibody efficiently coprecipitated the ^32^P-labelled HCV RNA (Fig. 2C, lane 4), whereas a Flag antibody did not coprecipitate HCV RNA (lane 3). As a positive control for the immunoprecipitation, an antibody against the eIF3A subunit was used (Fig. 2C, lane 2). The binding of Ago2 to the HCV 5′-UTR was specifically mediated by miR-122, since virtually no HCV RNA was precipitated with the Ago2 antibody in the presence of miR-124 (lane 5), a microRNA that does not confer HCV translation stimulation [12], [14], [30]. The immunoblot control shown in Fig. 2D confirms the recovery of Ago2 protein by the immunoprecipitation with the anti-Ago2 antibody in the presence of miR-122 as well as miR-124.

### The HCV 3′-UTR does not bind Ago2 protein

**Figure 3:**
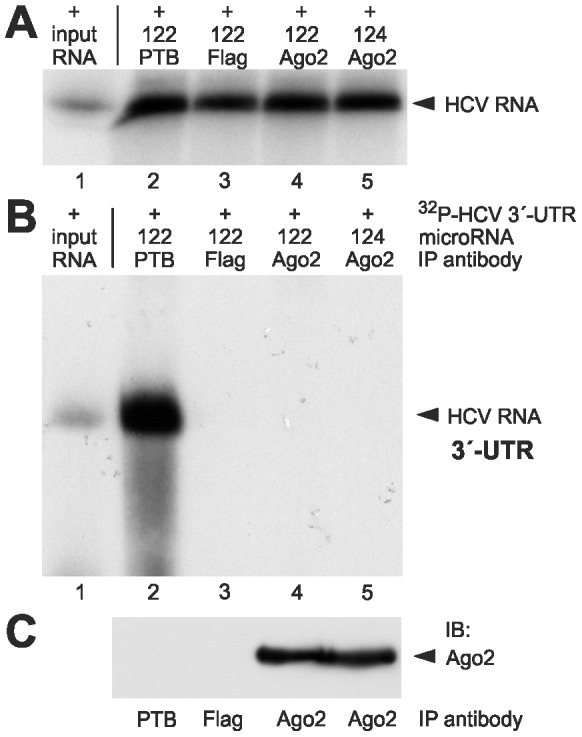
Ago2 protein does essentially not interact with the HCV 3′-UTR. (A-C) Analysis of ^32^P-labelled HCV 3′-UTR RNA after transfection into HeLa cells in the presence or absence of miR-122 or control miR-124 duplexes as indicated. The cells were lysed 6 h after transfection. (A) Aliquots of the cell lysate were used for RNA re-extraction to check the integrity of the input RNA prior to immunoprecipitation. The re-extracted radioactive HCV RNA was visualized after gel electrophoresis and autoradiography. A quantification of the RNA amounts in lanes 2–5 is shown in Fig. S1B. (B) Analysis of the HCV 3′-UTR RNA after immunoprecipitation with Ago2 antibodies. From the precipitate, RNA was re-extracted, and the radiolabelled HCV RNA was visualized after gel electrophoresis and autoradiography. In lane 1, the input RNA is shown (1∶100 dilution). In lane 2, an anti-PTB antibody was used as a positive control, and in lane 3, an anti-Flag antibody was used as a negative control. (C) Aliquots of the immunoprecipitates used in lanes 2–5 in (B) were checked for Ago2 in an immunoblot.

The HCV 3′-UTR contains a conserved miR-122 target consensus sequence (ACACUCC; see Fig. 1) in the otherwise variable region. Thus, we suspected that in the presence of miR-122 Ago2 may also bind to the HCV 3′-UTR sequence. However, we were not able to coprecipitate the ^32^P-labelled HCV 3′-UTR RNA with an anti-Ago2 antibody, neither in the presence of miR-122 nor in its absence (Fig. 3B, lanes 4 and 5). Again, an immunoblot control (Fig. 3C) confirms the recovery of Ago2 protein by the immunoprecipitation with the anti-Ago2 antibody. In this assay, an antibody directed against the polypyrimidine tract-binding protein PTB that binds to the poly(U/C)-tract of the 3′-UTR served as positive control for the coprecipitation, and this anti-PTB antibody precipitated large amounts of the HCV 3′-UTR RNA (lane 2). Thus, under the same experimental conditions that recovered large amounts of the HCV 5′-UTR RNA by the miR-122-mediated binding of Ago2 (compare Fig. 2C, lane 4) we could not detect miR-122-mediated binding of Ago2 protein to the HCV 3′-UTR.

### miR-122 recruits Ago1 to the HCV 5’-UTR

**Figure 4:**
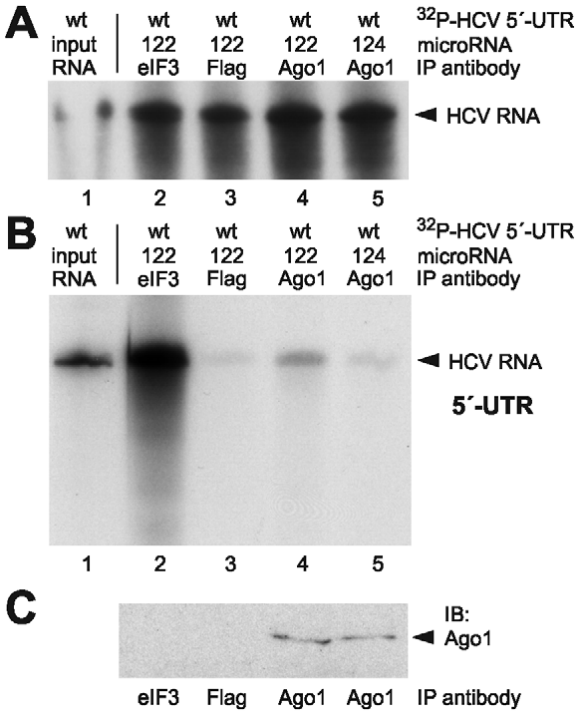
Ago1 protein interacts weakly with the HCV 5′-UTR. (A–C) Analysis of ^32^P-labelled HCV 5′-UTR RNA after transfection into HeLa cells in the presence or absence of miR-122 or control miR-124 duplexes as indicated. The cells were lysed 6 h after transfection. (A) Aliquots of the cell lysate were used for RNA re-extraction to check the integrity of the input RNA prior to immunoprecipitation. The re-extracted radioactive HCV RNA was visualized after gel electrophoresis and autoradiography. A quantification of the RNA amounts in lanes 2–5 is shown in Fig. S1C. (B) Analysis of the HCV 5′-UTR RNA after immunoprecipitation with Ago1 antibodies. From the precipitate, RNA was re-extracted, and the radiolabelled HCV RNA was visualized after gel electrophoresis and autoradiography. In lane 1, the input RNA is shown (1∶100 dilution). In lane 2, an anti-eIF3 antibody was used as a positive control, and in lane 3, an anti-Flag antibody was used as a negative control. (C) Aliquots of the immunoprecipitates used in lanes 2–5 in (B) were checked for Ago1 in an immunoblot.

There is indirect evidence that besides Ago2 also other members of the Ago-protein family are involved in miR-122 action on the HCV genome [33], [34]. Therefore, we checked whether there is a direct interaction between the HCV 5’-UTR RNA and Ago1. Ago1 was chosen because it is the most abundant Ago protein besides Ago2 in HeLa cells [35]. We performed a coimmunoprecipitation (as described before) using the rat-anti-Ago1 clone 4B8 [31]. Fig. 4 shows that the radioactively labeled HCV 5’-UTR was coprecipitated by the anti-Ago1 antibody slightly more efficiently in presence of miR-122 (lane 4) compared with miR-124 (lane 5) or with the anti-Flag antibody used as negative control (lane 3). Strikingly, the band we obtained from this experiment seems to be much weaker than the corresponding band in our Ago2 experiment (compare Fig. 2C, lane 4). However, we must take into account that this effect may at least in part arise from differences in Ago protein abundance since Ago2 represents about 62% of total Ago protein in HeLa cells, whereas Ago1 represents only 22% [35]. Moreover, the binding affinities of the antibodies to their respective antigens may be different (compare the immunoblots detecting Ago2 in Fig. 2D and Ago1 in Fig. 4C). Thus, we cannot exclude a functional role for Ago1 in HCV translation stimulation.

### Only miRNA duplex precursors of the correct length stimulate HCV translation

**Figure 5:**
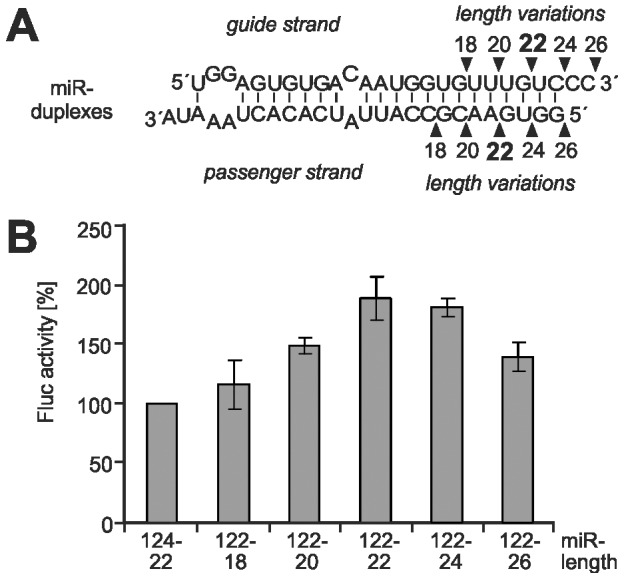
Length variation of the miR-122 duplex precursor impairs stimulation of HCV translation. (A) Structure of the miR-122 duplex (22 nucleotides) and its length variants. The base-pairing conditions at the thermodynamically less stable end (left side) and the 2 nucleotide 3′-overhangs at both ends were retained in all cases. (B) Stimulation of HCV reporter RNA translation in HeLa cells after co-transfection of the RNA duplexes shown in (A) as indicated. miR-124 was used as negative control, and its readout was set to 100%.

In order to investigate the functional implications of the binding of Ago proteins to the HCV 5′-UTR, we analyzed the exact length requirements for the miRNA duplex precursor substrates that are required for the stimulation of HCV translation. The monocistronic HCV reporter RNA (Fig. 1) was transfected into HeLa cells along with duplex miR-122 variants of different length (Fig. 5A). Fig. 5B shows that the wild type duplex miR-122 with 22 nt length stimulated HCV translation strongest, whereas shorter or longer duplex miR-122 variants stimulated to gradually lesser extents. In comparison to the addition of miR-124, the increase in translation efficiency with miR-122 variant duplexes was significant (p<0.05) for all length variants of 20 nts and more. In turn, when comparing the decrease in translation efficiency with miR-122 duplex precursors of lengths other than 22 nts with that of the original 22 nts duplex precursor, the decrease with the 18mer was clearly significant (p<0.01), while the decrease with the 26mer was almost significant (p = 0.0508).

These results suggest that the length of the miR-122 precursor is measured in the cell. The single-strand microRNA guide strand bound with its 5′-phophate in a pocket in the Ago MID domain and with its 3′-end in the PAZ domain ideally has a length of 22 nucleotides, with variations from 21 to 24 nts [36], [37], [38], [39]. This indicates that Ago proteins are involved in loading miR-122 to the HCV RNA and in translation stimulation.

### Insertions between the miR-122 binding sites and the IRES only slightly decrease translation stimulation by miR-122

**Figure 6:**
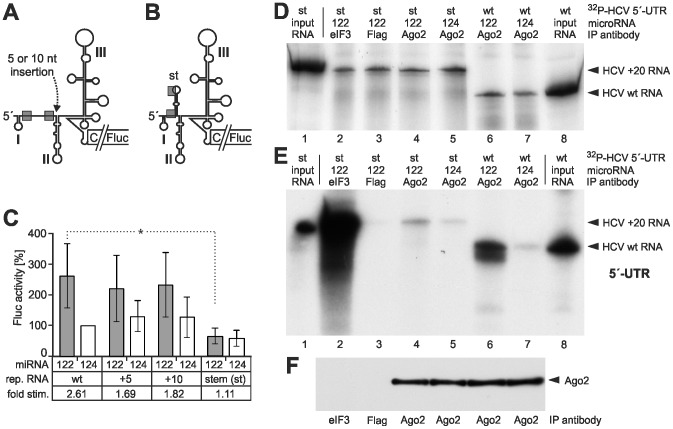
Influence of insertions between miR-122 target sites and IRES on miR-122-mediated stimulation of HCV translation. (A) In the HCV reporter RNA, 5 or 10 nucleotides (nt) were inserted between the second miR-122 binding site and the base of stem-loop II. (B) An insert between the second miR-122 target site and the stem-loop II was designed to mask the two miR-122 target sites in an additional stem (st). (C) Relative translation activities of the wild-type (wt) and mutant HCV reporter RNAs in HuH-7 cells. Additional ectopic miR-122 (or miR-124 as a control) was added as indicated. A capped and polyadenylated Renilla-Luciferase (Rluc) was co-transfected. The Rluc readouts were used for normalization of the Fluc readouts from the HCV reporter RNA. The expression of the HCV reporter RNA in the presence of the control miR-124 was set 100%. The p-value of the statistical difference is indicated for the stem insertion construct compared with the wt HCV reporter RNA (* = p<0.05). (D–F) Immunoprecipitation of Ago2-HCV 5′-UTR RNA complexes with the constructs shown in A and B. The experiments were performed essentially as in Fig. 2 B–D. (D) Examination of the radioactive HCV RNA re-extracted from the cell lysate prior to immunoprecipitation (as in Fig. 2B). A quantification of the RNA amounts in lanes 2–7 is shown in Fig. S1D. (E) Radiolabelled HCV RNA recovered by anti-Ago2 immunoprecipitation. In lanes 2–5, the HCV RNA with the additional stem (st) was used, in lanes 6–7, the wt HCV 5′-UTR RNA was used. In lane 1, the input RNA with the stem insert, in lane 8 the input wt HCV 5′-UTR RNA is shown (each in 1∶100 dilution). In lane 2, an anti-eIF3 antibody was used as a positive control. In lane 3, an anti-Flag antibody was used as a negative control. (F) Anti-Ago2 immunoblot control of the samples analyzed in (E).

The miR-122 target sequences are located in a fixed distance relative to each other and to the stem-loops I and II in virtually all HCV 5′-UTRs (see Fig. S2). Such a fixed distance between the stem-loops I and II might be important for various aspects of HCV replication. To test if the action of the Ago2-containing protein complexes tolerates insertions relative to the HCV IRES in order to mediate functional changes to the translation apparatus, we inserted stretches of 5 or 10 nucleotides between the second miR-122 target sequence and the base of stem-loop II (Fig. 6A). These insertions resulted in a negligible reduction in stimulation of HCV translation by ectopically added miR-122 in HuH-7 cells (Fig. 6C). Due to RNA fold predictions (not shown), the 5 nucleotide insertion is supposed to be single-stranded whereas the 10 nucleotide insertion may allow some base pairing. However, in any case single-stranded RNA inserts would allow for a certain flexibility of the flanking regions. Thus, the conservation of the HCV 5′-UTR sequences may primarily be important for other aspects of viral replication like RNA plus strand initiation [40], while the stimulatory action of the miRNP complex involving miR-122 and the Ago-proteins on IRES activity allows for a certain spatial flexibility.

### Masking the miR-122 binding sites in a stem-loop structure impairs Ago2 binding and translation stimulation by miR-122

In order to test if Ago2 is still recruited to a mutated HCV 5′-UTR in which the two miR-122 binding sites are largely masked, we inserted a sequence downstream of the second miR-122 seed target site that could largely base-pair with the upstream sequences containing the two miR-122 target sites (see Fig. 6B). With this mutant (stem), the translation efficiency in the HuH-7 cells was lower than with the wt 5′-UTR (Fig. 6C), suggesting that binding of the miR-122 which is contained endogenously in the HuH-7 cells to the masked miR-122 target sites was impaired. Moreover, also the stimulation of HCV translation by additionally added miR-122 was abolished (Fig. 6C, "stem"), indicating that also the ectopically added miR-122 could not bind to the masked miR-122 target sites. Accordingly, the results of a coimmunoprecipitation assay show that binding of Ago2 to the stem-loop insertion mutant is largely impaired compared with the binding of Ago2 to the wild type HCV RNA (Fig. 6E, compare lane 4 with lane 6), while the immunoblot control (Fig. 6F) confirms Ago2 recovery in the immunoprecipitation. The intensity of the HCV RNA signal with the Ago2 antibody in the presence of miR-122 is only slightly more than that obtained with the negative control miR-124 (lane 5). In contrast, by using an anti-eIF3 antibody in the coprecipitation large amounts of the radiolabelled HCV 5′-UTR RNA with the stem-loop insertion were recovered (lane 2). Analysis of the input RNA recovered prior to immunoprecipitation (Fig. 6D) and its quantitative analysis (Fig. S1D) show that different RNA amounts appear not to be the major reason for the differences in Ago binding. Thus, the binding of Ago2 protein to the mutant RNA is largely impaired by masking the two miR-122 binding sites in a stem-loop, suggesting that the microRNA in the miRNP complex is not able to invade a miRNA target site that is largely masked in a secondary structure. Moreover, the correlation of miR-122-dependent Ago2 binding and translation stimulation indicates that Ago2 is functionally involved in miR-122-mediated HCV translation stimulation.

### RNA stability contributes to HCV translation stimulation mediated by Ago2-miR-122 complexes

In order to find out if RNA stability may contribute to the stimulation of HCV translation in the presence of miR-122 binding, we used a RNA decay assay with two different reporter RNAs. The wt HCV 5′-UTR can bind miR-122 (irrespective if endogenously contained in the HuH-7 cells or ectopically added), whereas the stem mutant (st) cannot bind miR-122 because the miR-122 binding sites are masked by formation of a stem (see above). These two different reporter RNAs were transfected each in the presence of miR-122 or miR-124, respectively, into HuH-7 cells that contain moderate amounts of endogenous miR-122 [16]. After 3, 6 and 9 hours, the cells were harvested and luciferase activities measured. The resulting luciferase activities are a measure for the combination of both the inherent translational activity of each RNA molecule and the amount of RNA molecules involved in translation. Since equal amounts of RNA were transfected into the cells, different translation activities measured shortly after transfection indicate different inherent translational activities of the RNAs in question, while different rates of decay in translation activity at later time points would additionally indicate different rates of superimposed RNA decay.

**Figure 7:**
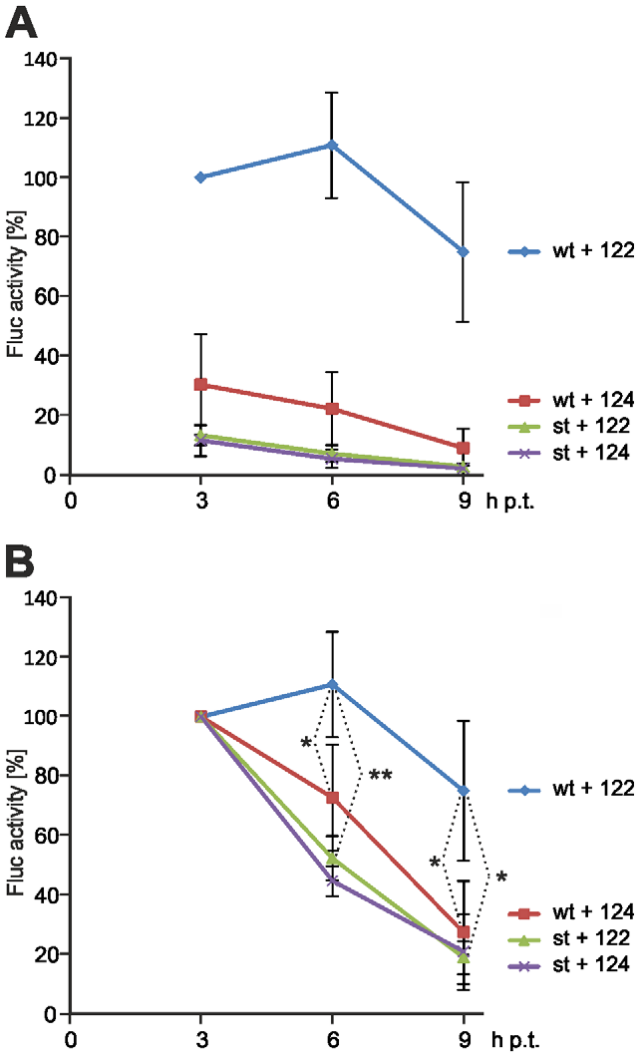
RNA decay time course. The HCV reporter RNA and the stem insertion mutant (st, see Fig. 6B) were transfected into HuH-7 cells. Additional ectopic miR-122 (or miR-124 as a control) was added as indicated. After the times indicated, the cells were lysed and Fluc activity determined. (**A**) Display of the Fluc activities with all readouts normalized to the wt HCV RNA in the presence of miR-122 after 3 h. All other readouts were normalized to this value to display the stimulation of translation by miR-122 at the 3 h time point and later time points. Error bars indicate standard deviations from four independent experiments. (**B**) The same readouts as shown in (A) but with the values obtained from expression of all combinations of HCV reporter RNAs and microRNAs at 3 h after transfection normalized to 100%. In this diagram, the differential decay in the presence of miR-122 or miR-124, respectively, is clearly obvious. * = p<0.05; ** = p<0.01.

The results of this assay (Fig. 7A) show that at 3 h after transfection, the translation efficiency of the wt HCV reporter RNA in the presence of miR-122 is about 3-fold higher than in the presence of miR-124. This indicates that the translation activity of the wt HCV reporter RNA is stimulated by the ectopically added miR-122 but not by added miR-124. In contrast, the translation activity of the stem mutant ("st" in Fig. 7A) is even lower than that of the wt HCV RNA in the presence of added miR-124. This indicates that the masked miR-122 binding sites in the mutated HCV 5′-UTR cannot bind even the moderate levels of miR-122 which are contained endogenously in the HuH-7 cells, whereas these endogenous miR-122 levels result in the slightly higher activity of the wt HCV reporter RNA in the absence of added miR-122.

Remarkably, in the presence of added miR-122 the translation activity of the wt HCV reporter RNA increases from 3 h to 6 h after transfection, while in the presence of added miR-124 the activity decreases, indicating that different decay of the HCV reporter RNAs may also add to the different translation activities. To compare the differences in the decay of translation activity more easily, we have plotted the translation activities of the RNAs after normalizing the readouts obtained at 3 h after transfection for all RNAs to 100% (Fig. 7B). With the wt HCV reporter RNA, the decay in translation activity from the value measured at 3 h after transfection was slower than that of the stem mutant even in the presence of added miR-124, indicating that even the endogenous miR-122 in the HuH-7 cells slowed down the decay of the HCV reporter RNA. In particular in the presence of ectopically added miR-122, the decay in translation activity of the wt HCV reporter RNA was significantly slower than that of the stem mutant and also slower than that of the wt RNA in the presence of miR-124.

## Discussion

In this study, we show that Ago2 protein is directly and efficiently recruited to the HCV 5′-UTR by miR-122 but not by an unrelated microRNA, indicating that direct base-pairing is used to guide a complex containing miR-122 and Ago2 to the HCV RNA. Preferentially miR-122 duplex precursors of 22 nucleotides in length are effective in stimulation of HCV translation. Both the stimulation of HCV translation by miR-122 and miR-122 mediated binding of Ago2 to the HCV 5′-UTR are impaired when the miR-122 binding sites in the HCV 5′-UTR are masked in a RNA secondary structure. The HCV reporter RNA translation efficiency decays faster in the absence of miR-122, indicating that also RNA stability contributes to overall translation stimulation by miR-122. Thus, binding of miR-122 and Ago protein appear to contribute to enhanced translation activity of the HCV 5′-UTR by two mechanisms, directly by the stimulation of translation and indirectly by inhibiting RNA decay.

Stimulation of HCV translation by miR-122 in HuH-7 and HeLa cells requires that duplex miR-122 precursors are co-transfected with the HCV RNA, as also shown previously [12], [30]. In contrast, in rabbit reticulocyte lysate - a widely used in vitro-translation system - only mature miR-122 guide strand stimulates HCV translation [30]. An inhibitory long-range RNA-RNA interaction can form between a sequence comprising part of the miR-122 binding sites and the sequence between them and a sequence in the core coding region [26], [27], [28]. Single-stranded miR-122 can displace this long-range interaction and switches the HCV IRES from a "closed" to an "open" conformation [29]. This switch probably makes the IRES more easily accessible for the ribosome and may also account for the rapid stimulation of ribosome binding to the HCV IRES by miR-122 in reticulocyte lysate [12]. Even though reticulocyte lysate contains Ago2 (D. Goergen and M.N., unpublished), our finding that duplex miRNA precursors are not effective in reticulocyte lysate [30] coincides with the finding that gene silencing in reticulocyte lysate requires pre-hybridization of single-stranded microRNA to the target mRNA [41]. Thus, the cellular machinery for processing microRNA precursors and loading the mature microRNA to an effector complex is not fully functional in reticulocytes, perhaps due to their high degree of specialization.

Our finding that in cells preferentially 22 nucleotide duplex miR-122 precursors are effective in HCV translation stimulation suggests the involvement of Ago proteins. Ago2-containing silencing complexes require duplex miRNA precursors of about 22 bp in length [36]. The duplex precursors are unwound, and the guide strand is transferred to an Ago protein that exposes the miRNA for hybridization with its mRNA target [42]. Thereby, Ago binds the miRNA guide strand with its 3′-end in the PAZ domain and the 5′-end between MID and PIWI domains [21], [22]. These spatial constraints restrict the length of the mature microRNA guide strand bound to Ago protein to a range from about 21 to 24 nts, with a preferred length of 22 nts [36], [37], [38], [39], [43], and the fact that miR-122 precursors of 24 nucleotides do not significantly differ in activity from those of 22 nucleotides is in accordance with the finding that Ago protein was found to frequently accept also miRNAs of 23 and 24 nucleotides in length [43]. Moreover, we have found previously [30] that the shortened 18mer variant of miR-122 used in Fig. 5 is essentially unable to stimulate HCV translation when provided as a duplex miRNA precursor in cells, whereas the same 18mer provided as a single stranded guide strand is able to displace an inhibitory long-range RNA-RNA interaction in the HCV RNA and by that stimulate translation in the reticulocyte lysate system (even better than the 22 nt miR-122 guide strand). Thus, the differences in translation efficiency observed with the miR-122 length variants are supposed to be due to inefficient Ago loading.

**Figure 8:**
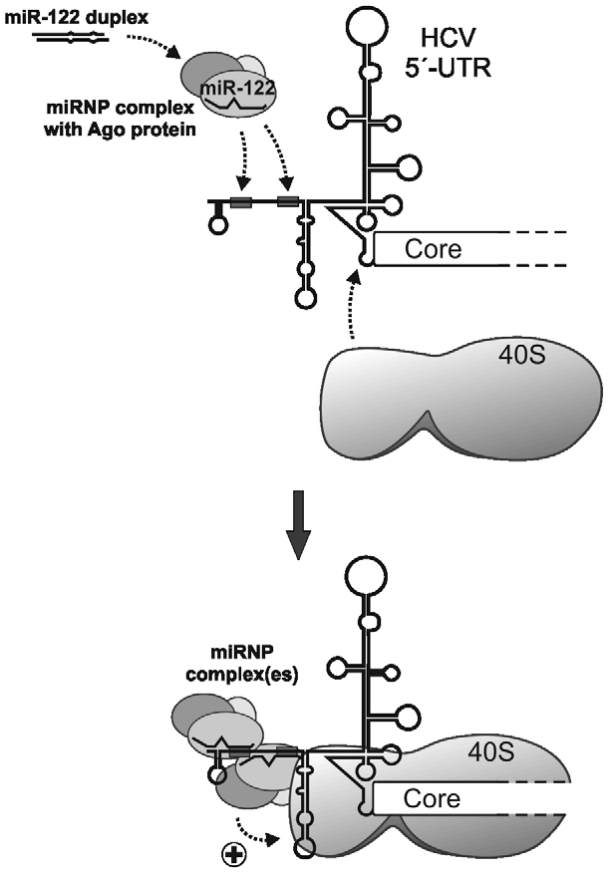
Hypothetical mechanism of the effect of miR-122 on HCV RNA stability and translation stimulation in cells. The mature miR-122 guide strand is processed from its duplex precursor and incorporated into functional microRNA-protein (miRNP) complexes. These complexes target one or two miR-122 target sites (grey boxes) in the HCV 5′-UTR and confer HCV RNA stability and translation stimulation (indicated by the (+)).

If the length of the duplex precursor is appropriate, the complex unwinds the duplex and loads the mature miR-122 guide strand to the target HCV RNA (Fig. 8), whereas miR-122 provided a priori as a single-stranded guide is not a substrate for this loading complex. We can only speculate if Ago2 remains associated with miR-122 after loading it to the HCV RNA. However, our observation that we could detect Ago binding after 6 hrs post transfection but not after 2 hrs (data not shown) indicates that in the presence of miR-122 Ago2 accumulates on the HCV RNA over time. The miR-122-mediated interaction of Ago2 with the HCV RNA appears to be considerably stable since it is not abolished during 3 hrs of immunoprecipitation from the cell extract under dilute conditions and is not washed off by 300 mM NaCl during the wash steps after IP. Moreover, the fact that single-stranded miR-122 is effective in reticulocyte lysate [30] implies that the single-stranded miR-122 guide strand (after being unwound from its duplex passenger) is able to bind the target HCV RNA. However, if Ago would be required only for duplex unwinding but not for the subsequent action on the HCV RNA, the single-stranded miR-122 guide strand should also be effective in cells. These observations suggest that the Ago2-containing complex may remain associated with the HCV RNA to enhance translation. When the HCV 5′-UTR binds to the small ribosomal 40S subunit, the region between the stem-loops I and II of the HCV 5′-UTR that bears the two miR-122 binding sites is located close to the head of the 40S subunit but exposed to the solvent [44], [45], leaving the miR-122 binding sites accessible for binding of the miR-122-Ago2 containing miRNP complex. We also do not know if the ribosomal 40S subunit or the miRNP complex bind first to the HCV 5′-UTR, but since the 40S subunit binds to the HCV IRES very tightly also in the absence of the miR-122 binding sequences [7] it can just be assumed that the 40S subunit binds first.

Indirect evidence for a role of protein components involved in miRNA precursor processing in HCV replication comes from RNA silencing studies. Silencing Drosha, DGCR8, Dicer as well as the Ago1-4 proteins (also called eIF2C1-4) resulted in reduced overall HCV replication and virus production [33]. Specific evidence for the involvement of Ago proteins in miR-122-mediated stimulation of HCV translation comes from two studies in which knock-down of Ago2 mRNA resulted in a reduction of HCV IRES directed translation efficiency by 25% [46] or 50% [34], respectively. In addition, also Ago1 may substantially contribute to HCV translation stimulation, since Ago1 mRNA knock-down also resulted in the reduction of miR-122-mediated translation stimulation [34]. This is consistent with our finding that also Ago1 is recruited to the HCV 5′-UTR.

Besides Ago and Dicer, also the RNA-binding protein TRBP which is a component of the RISC loading complex [42] may be a candidate protein which could be suspected to be involved in the miR-122-miRNP complex acting on the HCV RNA. Depletion of TRBP reduces miR-122-dependent HCV RNA accumulation when mature duplex miR-122 precursors are supplied [47], suggesting that TRBP is also a component of the miRNP complexes acting on the HCV RNA. Moreover, the switch from translation repression to translation activation by microRNA in the G_0_ phase of the cell cycle has been linked to enrichment of the FXR1 protein [24], [48]. Since the activity of the HCV IRES together with miR-122 stimulation is highest in the G_1_ and G_0_ phases of the cell cycle [14] - where the G_0_ phase may be the preferred state of metabolically active hepatocytes in the liver - FXR1 may also be a candidate involved in miR-122-miRNP action.

More and more evidence emerges that miR-122 has more than one function in the HCV replication cycle. Based on the finding that miR-122 stimulates HCV RNA accumulation in cells, it was originally proposed that HCV replication (more specifically, HCV RNA genome synthesis) is the object of miR-122 action in the viral life cycle [11]. Using a monocistronic reporter system [5] to avoid the collateral translation activation of the HCV IRES by additional IRES elements in cis [49], we have shown that also the stimulation of translation contributes to the positive effect of miR-122 on HCV propagation, notably not excluding other additional modes of miR-122 action [12], [14], [30]. This effect of miR-122 on HCV translation was confirmed by other groups [34], [46], [50]. However, translation stimulation was shown to be not fully sufficient to explain miR-122 action, since mutations in the miR-122 target sites have a much more pronounced effect on HCV virus yield than mutations in the IRES stem-loop IIId that have a quantitatively similar effect on mere translation [50]. While the elongation phase of HCV RNA synthesis was excluded to contribute to stimulation by miR-122 [51], the most likely simultaneous binding of two molecules of miR-122 with their seed sequences and also internal miRNA nucleotides to the 5′-end of the HCV RNA may result in a largely double-stranded RNA complex at the 5′-end of the HCV RNA [52].

The binding of miR-122 may mask the HCV RNA 5′-end, thereby protecting it from nucleolytic degradation. Indeed, the recruitment of miR-122 to the HCV 5′-UTR was shown to result in prolonged stability of the HCV RNA [53], [54]. These conclusions are supported by the results of our RNA decay assay (Fig. 7) which suggest that the binding of miR-122 and Ago protein to the HCV 5′-UTR results in elevated stability of the HCV RNA as well as in the stimulation of translation. However, we do not know why we - in accordance with the finding of Shimakami and coworkers [53], [54] - detected a contribution of RNA decay to the overall translation enhancement (Fig. 7), while we could not detect obvious differences in the RNA amounts when using the short HCV 5′-UTR RNAs for the detection of miR-122 dependent Ago protein binding (e.g., Fig. 2). We can only speculate if the sensitivity for RNases of the RNAs used may be different, perhaps due to the higher sensitivity of RNA which is actively translated or to the different length of the RNAs; the short 5′-UTR we used for the detection of Ago binding may be tightly covered by the ribosomal 40S subunit, eIF3 and the miR-122-Ago complexes. Also the finding that HCV NS5B, miR-122 and Ago2 (but not GW182) colocalize in replication complexes [55] is consistent with the idea that the miR-122-protein complex at the HCV RNÁs 5′-end may contribute to increased stability of the HCV RNA.

Even if HCV translation may be stimulated indirectly by enhanced stability of the RNA to be translated (i.e., by providing more template for translation), there is also evidence supporting the idea that the miR-122-Ago complexes are involved in directly stimulating translation. The exchange of the stem-loops III and IV of the HCV 5′-UTR by the related structures of Classical Swine Fever Virus (CSFV) resulted in a loss of translation stimulation by miR-122 [34]. This finding suggests that the miR-122-miRNP may specifically interact with the HCV IRES, and this interaction is impaired by a change in IRES structure and/or sequence. Moreover, the fraction of HCV 5′-UTR RNA which was bound to ribosomes was enhanced by the interaction with the microRNA after transfection into living HeLa cells [12]. These above findings suggest that HCV translation is stimulated not only by providing more template for translation after protection of the HCV against nucleolytic degradation but also by a direct interaction with the translation apparatus bound to the IRES element.

Taken together, Ago protein loads miR-122 to the two binding sites in the HCV 5′-UTR. Thereby, possibly both target sites in the HCV 5′-UTR are occupied with miR-122 miRNP complexes. These miRNP complexes then confer both enhanced stability of the HCV RNA by protecting its 5′-end against exonucleolytic degradation and the stimulation of translation (Fig. 8). We can only speculate if the interaction of the miR-122 miRNP complex associated with the first target site may be involved in protecting the HCV RNÁs 5′-end against degradation, and the miR-122 miRNP complex associated with the second miR-122 target site facilitates an overall increase in translation initiation efficiency by an interaction with the ribosome or associated factors.

## Materials and Methods

### Plasmids

Plasmid pHCV-374-Fluc-3′-UTR was derived from pHCV-Fluc-3′-UTR [5]. It contains a T7 promoter fused exactly to the HCV 5′-UTR (nucleotides (nts) 1-341), followed by nucleotides 342-374 (i.e., 11 codons) of the Core-coding sequence directly fused to the firefly luciferase (Fluc) gene and the entire HCV 3′-UTR (see Fig. 1) [5], [30]. For the insertion mutants, insertions of 5 or 10 nts (5’-TCGAG-3’ or 5’-TCTCGAGGTG-3’, respectively) were inserted between positions 43 and 44 between the second miR-122 target site and the left base of the IRES stem-loop II (see Fig. 6A). For insertion of the additional stem-loop masking the miR-122 target sites, an insertion of 20 nt (5’-GACTGATCTCGAGGTGGACT-3’) was placed inserted between positions 43 and 44 between the second miR-122 target site and the left base of the IRES stem-loop II (see Fig. 6B). This sequence can largely base-pair with the upstream miR-122 target sites. Plasmid pHCV-3′-UTR only was derived from plasmid pD12 [56]. Briefly, it contains downstream of a SP6 RNA polymerase promoter a 41 nt linker sequence followed by the HCV 3′-UTR sequence starting with the NS5B stop codon and ending with the 3′-terminal HCV nucleotide.

### RNA oligonucleotides

RNA oligonucleotides were supplied by biomers.net. The sequences were:

miR-122 mat, 5′-(PHOS)UGGAGUGUGACAAUGGUGUUUG-3′;

miR-122*, 5′-(PHOS)AACGCCAUUAUCACACUAAAUA-3′;

miR-124mat, 5′-(PHOS)UUAAGGCACGCGGUGAAUGCCA-3′;

miR-124*, 5′-(PHOS)GUGUUCACAGCGGACCUUGAUU-3′;

The sequences of other length variants of miR-122 are shown in Fig. 1B. Duplexes were formed between the guide (mat) and its complementary passenger strand (*).

### RNA synthesis

DNA oligonucleotides for template PCR were supplied by biomers.net. The sequences were:

HCMV-4986 for, 5′-CCAATAGGCCGAAATCGGCAAAATCCC-3′;

HCV3X rev, 5′-ACATGATCTGCAGAGAGGCCAG-3′;

HCV-FL 334-363 rev, 5’-CGTGCACCATGAGCACGAATCCTAAACCTC-3′;

HCV-FL 360-386 rev, 5’-GGCGTCTTCCATGGTTTTTCTTTGAGG-3′;

3′-UTR fwd, 5’-CGTCAGAAGCTAGCGATTTAGGTG-3′.

Primers HCMV-4986 for and HCV3X rev were used to generate the PCR template for the complete HCV IRES-Fluc-3′-UTR RNA from plasmid pHCV-374-Fluc-3′-UTR [30]. The HCV 5′-UTR used in Fig. 2 was transcribed from a PCR fragment generated with primers HCMV-4986 for and HCV-FL334-363 rev from plasmid pHCV-374-Fluc-3′-UTR. The resulting RNA of 363 nts starts at HCV position 1 and ends at position 363 in the core coding sequence. The template for the HCV 5′-UTR RNAs used in Fig. 3, 4 and 5 was made with primers HCMV-4986 for and HCV-FL 360-386 rev from plasmid pHCV-374-Fluc-3′-UTR. The resulting RNAs starts at HCV position 1, extends to position 374 in the core coding sequence and contains 12 additional nts of Fluc coding sequence. The transcripts have a length of 386 nts in the case of the wt HCV RNA and of 406 nts in the case of the stem-loop insertion RNA used in Fig. 5. Primers 3′-UTR fwd and HCV3X rev were used to generate the "3′-UTR only" RNA from plasmid pHCV-3′-UTR only. Transcription with SP6 RNA polymerase then resulted in a HCV 3′-UTR RNA of 265 nts.

The above templates for HCV reporter RNAs were amplified by PCR from the respective plasmids, the PCR fragments purified by the Roche GFX kit or by proteinase K digestion, phenol/chloroform extraction and ethanol precipitation, dissolved in water and used for transcription using T7 or SP6 RNA polymerase. In vitro transcription was performed in the presence of 500 µM NTPs. To generate radioactively labelled RNA, in vitro transcription was performed using T7 RNA polymerase in the presence of 500 µM ATP, GTP and CTP, 100 µM UTP and 0.625 µM or 0.5 µM [α-^32^P] UTP (800 Ci/mmol; PerkinElmer). The integrity of unlabelled RNAs was checked by agarose gel electrophoresis. Aliquots of the radiolabelled RNAs were run on 7 M urea/6% polyacrylamide gels and checked by autoradiography, and RNA concentrations were determined by gel images and photometric analyses. The concentrations of the labelled RNAs obtained in the in vitro-transcription reactions were calculated from the amount of the limiting radiolabelled nucleotide used in the in vitro-transcription and from the estimated efficiency of label incorporation (% nucleotides incorporated into the RNA versus % non-incorporated nucleotides). Unlabelled RNAs were purified using the RNAeasy protocol from Qiagen.

### Transfection of cell lines and reporter assays

For translation of HCV reporter RNAs in human hepatoma cells (HuH-7) or HeLa cells, cells were seeded in 24 well plates with 60% confluency 24 h before transfection. Transfection of 400 ng HCV reporter RNA and 400 ng miR duplexes per well (plus 20 ng of capped and polyadenylated co-transfected Rluc reporter RNA, if appropriate) was performed using Lipofectamine 2000 (Invitrogen). 4 h after transfection, the cells were washed in phosphate buffered saline (PBS). 150 µl passive lysis buffer (Promega) was added to each well, and cells were lysed by gentle agitation. Nuclei were removed by centrifugation, and 20 µl of the supernatant was used for measuring Fluc activity. Expression values were corrected for variations in cell density by measuring respiratory chain activity in a WST-1 assay (Roche) according to the manufactureŕs protocol with the exception that the WST-1 reagent was diluted 1∶50 and incubated for 30 min on the cells [57]. In addition, the translation efficiency of the co-transfected capped and polyadenylated Renilla Luciferase (Rluc) mRNA was measured and used for well-to-well normalization [57].

### Immunoprecipitations, RNA stability controls and western blots

1-4×10^8^ HeLa cells were seeded in 9 cm plates and transfected on the next day (when they were approximately 90% confluent) with 3 µg ^32^P-labelled HCV reporter RNA and 3 µg of active microRNA (i.e. 6 µg of miR duplex) using Lipofectamine 2000. After 6 h incubation, the cells were harvested [31]. For RNA stability controls, total RNA was isolated from an aliquot of the cell extract by proteinase K digestion, phenol/chloroform extraction and ethanol precipitation, and the radiolabelled HCV RNA was visualized after gel electrophoresis on 7 M urea/6% polyacrylamide gels and autoradiography. From the rest of the cell lysates, the immunoprecipitation (IP) was performed essentially according to the protocol of Beitzinger et al. (2007) for 3 hrs using the Ago2 monoclonal antibody 11A9 [32] or the Ago1 monoclonal antibody 4B8 [31] and Protein G beads (New England Biolabs, NEB). For controls, mouse anti-Flag M2 (Sigma), rabbit polyclonal anti-eIF3A (Abcam) and goat anti PTBP1 (Abcam) antibodies were used. Protein A beads (NEB) were used for the eIF3 antibodies. After the IP and four washing steps, aliquots of the beads were taken for western blots. Anti-Ago2 western blots were performed as described [32]. From the rest of the beads, RNA was isolated [31] and analyzed by gel electrophoresis on 6% polyacrylamide/7 M urea gels and autoradiography.

## Supporting Information

Figure S1 **Quantification of the RNA input controls.** (A) Densitometric scan of the RNA amounts in Fig. 2B, lanes 2–5. The value of the first sample was set to 100%. (B) Densitometric scan of the RNA amounts in Fig. 3A, lanes 2–5. (C) Densitometric scan of the RNA amounts in Fig. 4A, lanes 2–5. (D) Densitometric scan of the RNA amounts in Fig. 6D, lanes 2–7.
(TIF)

Figure S2 **Conservation of the 5′-terminal sequences of the HCV 5′-UTR among different HCV isolates.** The sequence comparison shows the sequences of various HCV genotypes from nucleotide position 1 up to the first 7 nucleotides of the stem-loop (SL) II (nucleotide No. 50 in genotype 1b). Strain number, accession number and in some cases the name of the isolate (in parentheses) are given. Conserved residues are highlighted by an asterisk (bottom line). The highly conserved miR-122 seed target consensus sequences are marked in red, and the stem-loops I and II are indicated on the top.
(TIF)
