# Correction: microRNA-122 Dependent Binding of Ago2 Protein to Hepatitis C Virus RNA Is Associated with Enhanced RNA Stability and Translation Stimulation

**DOI:** 10.1371/journal.pone.0160132

**Published:** 2016-07-29

**Authors:** K. Dominik Conrad, Florian Giering, Corinna Erfurth, Angelina Neumann, Carmen Fehr, Gunter Meister, Michael Niepmann

A funding grant was omitted from the Financial Disclosure of the published article. The complete, correct, Financial Disclosure is: This work was supported by the Deutsche Forschungsgemeinschaft, Germany (DFG) (SFB 535, Ni 604/1-1 and /2-1, GRK 1384, SFB 1021) and the Universitätsklinikum Giessen und Marburg GmbH (KOOPV). The funders had no role in study design, data collection and analysis, decision to publish, or preparation of the manuscript.

## References

[1] Conrad KD, Giering F, Erfurth C, Neumann A, Fehr C, Meister G, et al (2013) microRNA-122 Dependent Binding of Ago2 Protein to Hepatitis C Virus RNA Is Associated with Enhanced RNA Stability and Translation Stimulation. PLoS ONE 8(2): e56272 doi: 10.1371/journal.pone.0056272 23405269 10.1371/journal.pone.0056272 PMC3566042

