# Supplementary figures and images for: microRNA-122 Dependent Binding of Ago2 Protein to Hepatitis C Virus RNA Is Associated with Enhanced RNA Stability and Translation Stimulation
Source: PLoS One. 2013 Feb 6;8(2):e56272. doi: 10.1371/journal.pone.0056272 (PMC3566042; doi:10.1371/journal.pone.0056272)

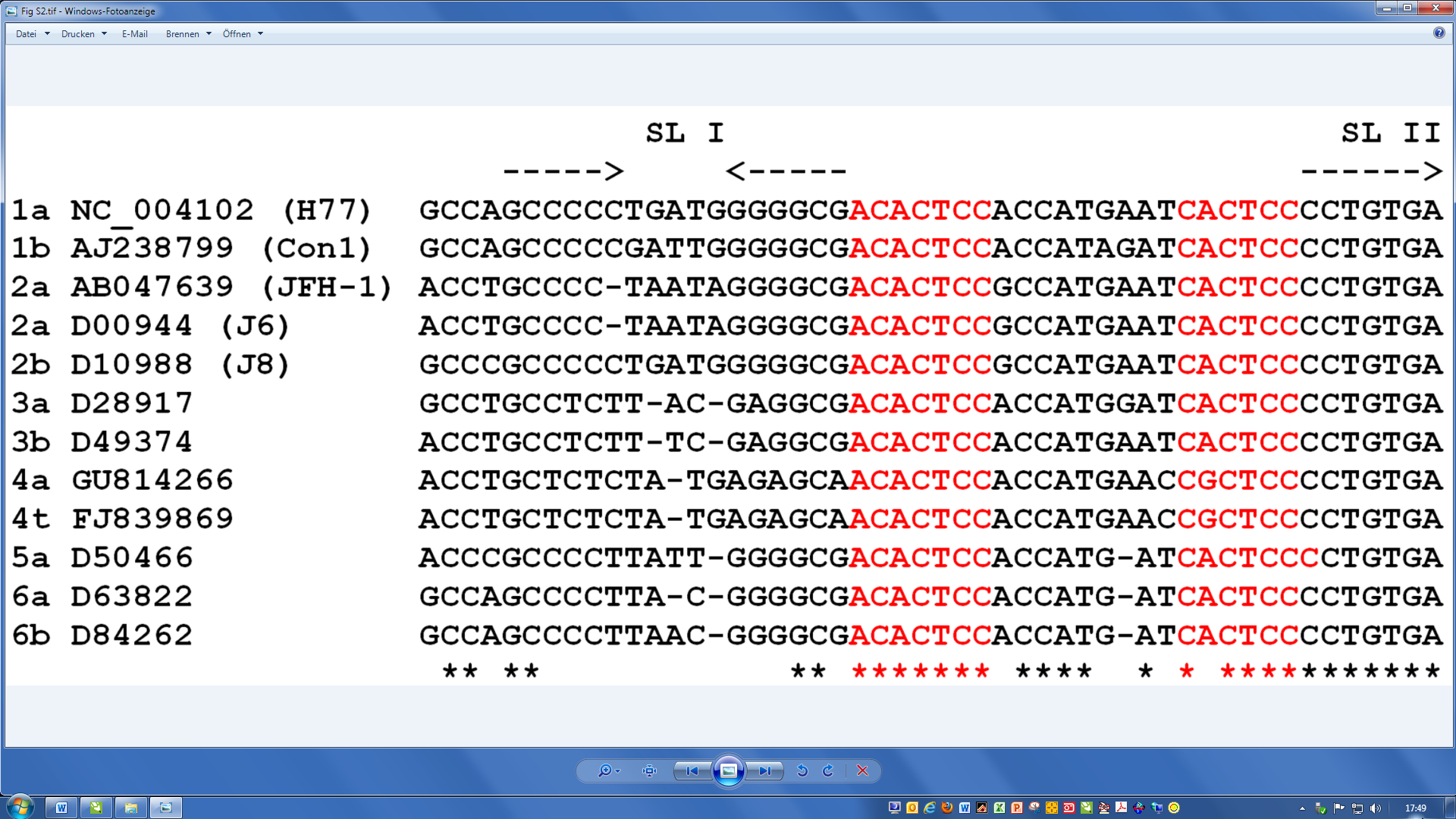

Supplement: Figure S2 — Conservation of the 5′-terminal sequences of the HCV 5′-UTR among different HCV isolates. The sequence comparison shows the sequences of various HCV genotypes from nucleotide position 1 up to the first 7 nucleotides of the stem-loop (SL) II (nucleotide No. 50 in genotype 1b). Strain number, accession number and in some cases the name of the isolate (in parentheses) are given. Conserved residues are highlighted by an asterisk (bottom line). The highly conserved miR-122 seed target consensus sequences are marked in red, and the stem-loops I and II are indicated on the top. (TIF) [file pone.0056272.s002.tif]
